# Field evaluation of specific mycobacterial protein-based skin test for the differentiation of *Mycobacterium bovis*-infected and Bacillus Calmette Guerin-vaccinated crossbred cattle in Ethiopia

**DOI:** 10.1111/tbed.14252

**Published:** 2021-08-19

**Authors:** Berecha Bayissa, Asegedech Sirak, Aboma Zewude, Adane Worku, Balako Gumi, Stefan Berg, R. Glyn Hewinson, James L. N. Wood, Gareth J. Jones, ETHICOBOTS consortium, H. Martin Vordermeier, Gobena Ameni

**Affiliations:** 1 Aklilu Lemma Institute of Pathobiology Addis Ababa University, Addis Ababa, Ethiopia; 2 Vaccine Production and Drug Formulation Directorate, National Veterinary Institute, Bishoftu, Ethiopia; 3 National Animal Health Diagnostic and Investigation Centre, Sebeta, Ethiopia; 4 Ethiopian Public Health Institute, Addis Ababa, Ethiopia; 5 Department of Bacteriology, Animal and Plant Health Agency, New Haw, Addlestone, Surrey, UK; 6 Aberystwyth University, Ceredigion, UK; 7 Department of Veterinary Medicine, University of Cambridge, Cambridge, UK; 8 Department of Veterinary Medicine, College of Food and Agriculture, United Arab Emirates University, Al Ain, United Arab Emirates

**Keywords:** BCG vaccination, bovine tuberculosis, crossbred cattle, DIVA skin test, specific mycobacterial proteins

## Abstract

Bovine tuberculosis (bTB) challenges intensive dairy production in Ethiopia and implementation of the test and slaughter control strategy is not economically acceptable in the country. Vaccination of cattle with Bacillus Calmette–Guerin (BCG) could be an important adjunct to control, which would require a diagnostic test to differentiate *Mycobacterium bovis* (*M. bovis*)-infected and BCG-vaccinated animals (DIVA role). This study describes an evaluation of a DIVA skin test (DST) that is based on a cocktail (DSTc) or fusion (DSTf) of specific (ESAT-6, CFP-10 and Rv3615c) *M. bovis* proteins in Zebu–Holstein–Friesians crossbred cattle in Ethiopia. The study animals used were 74 calves (35 BCG vaccinated and 39 unvaccinated) aged less than 3 weeks at the start of experiment and 68 naturally infected ‘TB reactor’ cows. Six weeks after vaccination, the 74 calves were tested with the DSTc and the single intradermal cervical comparative tuberculin (SICCT) test. The TB reactor cows were tested with the DSTc and the SICCT test. Reactions to the DSTc were not observed in BCG-vaccinated and unvaccinated calves, while SICCT test reactions were detected in vaccinated calves. DSTc reactions were detected in 95.6% of the TB reactor cows and single intradermal tuberculin positive reactions were found in 98.2% (95% confidence interval, CI, 92.1–100%). The sensitivity of the DSTc was 95.6% (95% CI, 87.6–99.1%), and significantly (*p* < .001) higher than the sensitivity (75%, 95% CI, 63.0–84.7%) of the SICCT test at 4 mm cut-off. DSTf and DSTc reactions were correlated (*r* = 0.75; 95% CI = 0.53–0.88). In conclusion, the DSTc could differentiate *M. bovis*-infected from BCG-vaccinated cattle in Ethiopia. DST had higher sensitivity than the SICCT test. Hence, the DSTc could be used as a diagnostic tool for bTB if BCG vaccination is implemented for the control of bTB in Ethiopia and other countries.

## 1 | INTRODUCTION

In Ethiopia, intensive dairy farms that raise genetically improved dairy cows have been established around cities and towns (Ameni et al., 2018; Mekonnen et al., 2019) to address national nutritional needs. The development of the dairy sector has been constrained by the emergence of diseases associated with intensification, including bovine tuberculosis (bTB) (Ameni et al., 2007; Firdissa et al., 2012). bTB is an endemic disease in Ethiopia (reviewed by Sibhat et al., 2017) and the disease is affecting livestock production through reduction of productivity and trade restrictions (OIE, 2018; Tschopp et al., 2012). As a zoonotic disease, bTB poses a public health threat, especially in low- and middle-income countries (LMICs) like in Ethiopia (Ashford et al., 2001).

Many developed countries have controlled bTB in their livestock populations using detection and slaughter of reactor animals (Buddle et al., 2013; Schiller et al., 2010). However, in many LMICs, the implementation of such control is economically and societally unacceptable and different control strategies need to be considered. Vaccination is an alternative control strategy and BCG is the only currently available vaccine. Research trials evaluating the efficacy of BCG vaccination against *M. bovis* infection in cattle have demonstrated promising results (Vordermeier et al., 2009; Ameni et al., 2010; Ameni et al., 2018; Vordermeier et al., 2016a ; reviewed by Buddle et al., 2018). However, BCG vaccination sensitizes vaccinated animals to react to tuberculin-based tests, such as single intradermal cervical comparative tuberculin (SICCT) or single intradermal tuberculin (SIT) tests (Vordermeier et al., 2001; Whelan et al., 2010) as BCG was originally derived from *M. bovis*, compromising their specificity. The current standard for diagnosis of TB in cattle measures cell-mediated immune response to intradermal injection of tuberculin. The SIT test is performed by injecting 0.1 ml of 3000 IU of bovine purified tuberculin (PPD-B) into the skin of the mid-cervical region or in the base of the tail, while the SICCT test entails simultaneous injection of both PPD-B and avian PPD (PPD-A) side-by-side into the skin of the neck for discriminating animals infected with *M. bovis* and those sensitized with *M. avium* complex or environmental non-tuberculous mycobacteria (reviewed by de la Rua-Domenech et al., 2006). However, both PPD-B and PPD-A are poorly defined antigens resulting in suboptimal sensitivity and inconsistent performance (Bezos et al., 2014; Buddle et al., 2009; Schiller et al., 2010). Therefore, there is a need for a diagnostic test that differentiates *M. bovis* infected from BCG-vaccinated animals (DIVA role).

Significant progress has been made applying defined antigens as DIVA tests for cattle (Jones et al., 2012; Srinivasan et al., 2019; Vordermeier et al., 1999; Vordermeier et al., 2002; Vordermeier et al., 2011; Vordermeier et al., 2016b), using mycobacterial antigens present in field strains of *M. bovis* but absent in the BCG vaccine. These antigens include early secretory antigen target-6 kDa (ESAT-6) and culture filtrate protein-10 kDa (CFP-10) (Pollock & Anderson, 1997; Vordermeier et al., 1999; Vordermeier et al., 2001). Their encoding genes are located within the region of difference 1 (RD1) of the *M. bovis* genome, a region was deleted from all BCG strains (Garnier et al., 2003; Gordon et al., 1999). As a result, T-cells of the BCG-vaccinated and/or non-infected cattle do not recognize ESAT-6 and CFP-10. However, the use of the cocktail of these two antigens showed a lower capacity in detecting infected animals compared with tuberculin-based tests (Sidders et al., 2008; Vordermeier et al., 2011). In attempts to overcome this limitation, the antigen Rv3615c was discovered to be a useful additional DIVA antigen to complement ESAT-6 and CFP-10 (Sidders et al., 2008).

This protein cocktail of Rv3615c, ESAT-6 and CFP-10 has previously been evaluated as a blood and skin test reagent, mainly in *Bos taurus* breeds, such as Holstein–Friesians (Casal et al., 2012; Vordermeier et al., 2016b) but not in zebu cattle or cross-breeds between zebus and taurine cattle. In the present study, the protein cocktail-based DIVA skin test (DSTc), as well as a fusion protein (DSTf) of the same antigens, were evaluated in Zebu–Holstein–Friesian crossbreed cattle under field condition.

## 2 | MATERIALS AND METHODS

### 2.1 | Study animals and husbandry

The study was conducted on Holstein-Friesian x Zebu crossbred calves and cows. The calves were all male and recruited from bTB free dairy farms within 2 weeks of age. Upon arrival at our animal facility, the calves were screened by the whole blood interferon-gamma release assay (IGRA) to demonstrate freedom from infection (data not shown). The cows (herein known as TB reactors) were recruited from a bTB-positive herd and tested positive for bTB upon recruitment by both IGRA and SICCT. The naïve calves and the TB reactor cows were kept in separate barns at the National Animal Health Diagnostic and Investigation Center at the Sebeta, Ethiopia. The calves were fed on pasteurized partially skimmed milk, hay and concentrate. The TB reactor cows were fed on hay and concentrate. Both the calves and the TB reactor cows were watered ad libitum.

### 2.2 | Study design, plan and setting

This is a cross-sectional study in which the performance of the DSTc was evaluated in comparison with the SIT and SICCT tests. First, the diagnostic specificity of DSTc was tested on 74 calves. The calves were randomly assigned into BCG vaccinated and control groups using a lottery method. Accordingly, 35 calves were vaccinated subcutaneously by 1 × 10^6^ CFU of BCG Sofia (InterVax Ltd, Toronto, ON, Canada) at 2 weeks of age, while the remaining 39 were kept unvaccinated. Relative low dose of 10^4^–10^6^ CFU has demonstrated to be efficacies in inducing protective immunity (Vordermeier et al., 2016b). After 6 weeks post-vaccination, both the vaccinated and unvaccinated calves were tested by the DSTc and SICCT tests. The sensitivity was tested on 68 TB reactor cows using both the DSTc and SICCT tests. In addition to the DSTc, an additional study was undertaken to assess the diagnostic performance of the recombinant fusion protein of ESAT-6, CFP-10 and Rv3615c as a DIVA skin test (DSTf). After 12 months of the previous trial, only 30 of the 68 TB reactor cows were left as the other 38 were culled due to shortage of logistics of keeping them for a longer time. Hence, these 30 TB reactor cows were used for comparison of the DSTf with the DSTc and SICCT test; they were tested simultaneously with intradermal injection of DSTf and DSTc on one side of the neck, while SICCT was applied on the other side of the neck of the study animals.

### 2.3 | Antigens

The cocktail protein-based DST (DSTc) consisted of the ESAT-6, CFP-10 and Rv3615c antigens of *M. tuberculosis* and *M. bovis*. The individual recombinant proteins were produced by Lionex GmbH, Braunschweig, Germany. Briefly, histidine-tagged recombinant proteins were expressed in *Escherichia coli* (*E. coli*), purified by nickel affinity chromatography, re-folded against 10 mM NH_4_HCO_3_ (pH 8.0) and lyophilized. Western blots demonstrated a positive reaction when using anti-histidine tag and/or protein-specific antibodies, and no reaction with anti-*E. coli* antibody. Purity was assessed at > 95% using SDS-PAGE and densitometry. When preparing the DSTc, equal amounts of each freeze-dried protein were combined in a PBS solution containing 100 *μ*g/ml of each protein (300 *μ*g total protein/ml). The DSTc solution was stored at −80°C until needed. The DSTf reagent was produced by Lionex following the same approach, with the exception that it was supplied as solution (300 *μ*g total protein/ml) after buffer exchange against phosphate buffered saline (PBS; pH 7.4) and stored at 4°C until being used.

### 2.4 | Protein-based DIVA skin test

All study animals (BCG vaccinated and control calves; bTB-positive cows) were injected intradermally with 0.1 ml DSTc (30 *μ*g total protein per dose) into the middle of the right side of the neck. For the comparison between the DSTc and DSTf, 0.1 ml DSTc (30 *μ*g protein per dose) was injected 10 cm below the crest and 0.1 ml DSTf was injected 12 cm below DSTc on a vertical line. Skin thicknesses were measured before inoculation and at 72 h post inoculation. The measurements were done by the same operator using the same digital caliper in every testing. Results are expressed as the difference in skin-fold thickness (in millimetre) before administration of the antigens and 72 h post administration. Skin reaction was considered positive if the increase in skin thickness at the DSTc or DSTf site was greater than or equal to 2 mm (Casal et al., 2012; Vordermeier et al., 2016b).

### 2.5 | Single intradermal cervical comparative tuberculin test

The SICCT test was performed on the left side of the study animals in the middle of the neck. After preparation of the injection site, 0.1 ml PPD-A (3000IU/ml; Prionics, Lelystad, The Netherlands) was inoculated 10 cm below the crest and the same volume of PPD-B (2500IU/ml; Prionics) was injected at a site 12 cm apart from PPD-A injection site in vertical line in reactor cows. The skin thicknesses were measured just before injection and at 72 h post injection by the same operator using the same digital caliper, and the results were presented as change in skin thickness (mm) between the two readings. In case of the SIT test, skin reaction was defined as positive when the increase of skin thickness at PPD-B site was greater than or equal to 4 mm, otherwise considered as negative. For the SICCT test, the differences in the increase of skin thickness at the bovine and avian PPD injection sites were considered. An animal was considered to be positive when the increase in skin thickness at the bovine PPD site was greater than the increase in skin thickness at the site of the avian injection by at least 4 mm. If the differential increases between the two sites were equal to or less than 1 mm, or between 1 and 4 mm, the animal was considered negative or doubtful, respectively (OIE, 2018).

### 2.6 | Data analysis

Data analysis was performed using Prism 8 (GraphPad Software). The skin-fold thickness increase was summarized using median and 95% confidence interval of median (95% CI) after assessment of normality of the data. Wilcoxon matched-pair signed rank test was performed for comparison of skin reactions induced by two defined antigens while using the Friedman test (repeated measures non-parametric analysis of variance) with Dunn’s multiple comparison test for more than two defined antigens. In addition, Spearman rank test was used for evaluation of the correlation of the degree of skin thickness induced by different antigens. A comparison of the DSTc relative sensitivity was scrutinized using the Fisher’s exact test. Kappa test was made to evaluate the diagnostic agreement between the DSTc and that of the SICCT or SIT test. In all cases, a 95% CI and a significant level of 5% were used to express statistical significance.

## 3 | RESULTS

### 3.1 | Performance of DSTc as a DIVA test in vaccinated and unvaccinated calves

**FIGURE 1:**
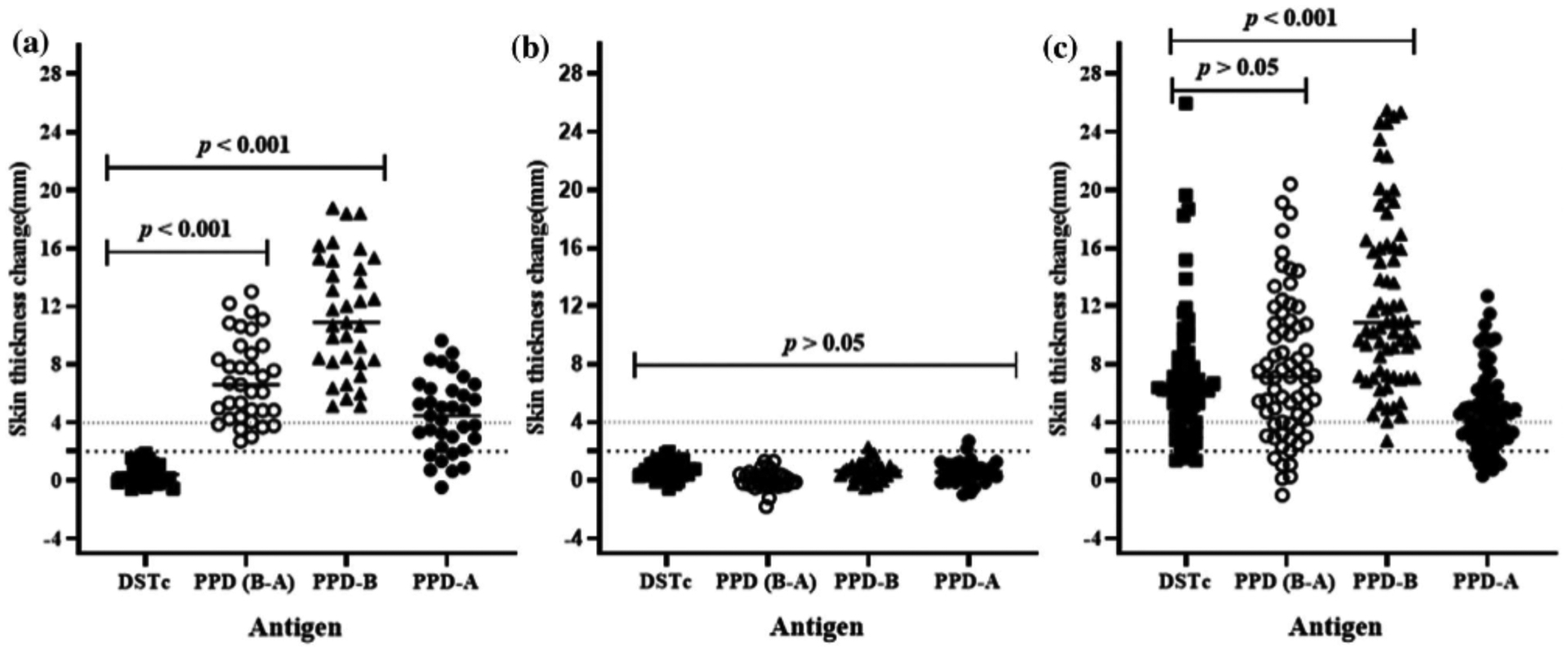
Skin thickness in response to DSTc, PPD-B, PPD-A and PPD-(B-A) in 35 BCG-vaccinated calves (Panel a), in 39 non-vaccinated naïve calves (Panel b) and in 68 naturally infected reactor cattle (Panel c). Individual animal skin thickness change (millimetre) between the pre- and post-skin test readings was represented by solid squares for DSTc, open circles for PPD (B-A), solid triangles for PPD-B and solid circles for PPD-A with a horizontal line providing the median change of respective defined antigen. The statistical difference in skin reaction was determined using non-parametric Friedman test with Dunn’s multiple comparison test. There was a significant difference (*p* < .001) between DSTc and tuberculin in causing skin reaction in the BCG-vaccinated calves, while similar skin reaction (*p* > .05) was developed following inoculation of all defined antigens in naïve calves. Significantly (*p* < .001) stronger skin response in naturally infected reactor cattle was also developed following PPD-B intradermal injection compared to the outcome of DSTc. The dashed horizontal lines at 2 and 4 mm are the cut-offs used for DSTc, and PPD-(B-A) and PPD-B, respectively

Six weeks post-vaccination, the BCG-vaccinated (*n* = 35) and unvaccinated control (*n* = 39) calves were skin tested with the DSTc and avian and bovine PPD (PPD-A, PPD-B) (Data S1). All BCG vaccinated calves were DSTc negative (Figure 1a, median of increase in skin thickness: 0.40 mm, 95% CI = 0.05–0.89). In contrast, all of the vaccinated calves responded to PPD-B and were SIT positive (Figure 1a, median reaction sizes PPD-B: 10.91 mm, 95% CI = 8.50–13.67). Furthermore, 82.9% (95% CI = 65.7–92.4) of the vaccinated calves were positive for the SICCT test (Figure 1a, median PPD-B minus PPD-A: 6.60 mm, 95% CI = 4.87–7.85). Comparative analysis indicated that the skin thickness induced by DSTc in calves was significantly lower than the skin thickness induced either by PPD-B (*p* < .001) or by PPD-A (*p* < .001). These data demonstrated the superior specificity of DSTc compared to SIT or SICCT. Therefore, the DSTc demonstrated its DIVA utility in crossbred cattle in Ethiopia. As observed with the BCG-vaccinated calves, none of the unvaccinated calves showed a skin reaction difference of 2 mm or higher and they were, therefore, classified as DSTc negative (Figure 1b; median reaction size: 0.72 mm, 95% CI = 0.33–0.99). Similarly, none of the unvaccinated calves were positive either in the SIT (median reaction size 0.64 mm, 95% CI = 0.30–0.90) or the SICCT test (median reaction size: 0.57 mm, 95% CI = 0.32–0.85; Figure 1b).

### 3.2 | Diagnostic performance of DSTc in bTB-infected cows

The diagnostic sensitivity of the DSTc was tested in 68 naturally infected TB reactor cows from a confirmed bTB-positive herd (Data S1). The recorded median of skin thickness increase was 6.25 mm (95% CI = 5.30–6.76) at the DSTc injection site, while the median skin thicknesses were 10.82 mm (95% CI = 9.55–13.63) at PPD-B and 4.48 mm (95% CI = 3.59–5.03) at PPD-A sites. The result indicated that the median increase of skin thickness to DSTc injection was significantly lower (*p* < .001) than the median in skin thickness at the injection site of PPD-B (Figure 1c). On the other hand, by considering the SICCT results, the median of the differences in skin thickness at the injection site of PPD-B and the injection site at PPD-A was 7.10 mm (95% CI = 5.54–8.40), which did not statistically (*p* > .05) differ from the median skin thickness at the injection site of DSTc. Thus, there was no difference in thicknesses induced by the DSTc and SICCT tests.

As summarized in Table 1, out of 68 animals, 65 were positive to the DSTc, resulting in a relative sensitivity of 95.6% (95% CI = 86.9–98.6%). Similarly, the relative sensitivity of SIT was 98.2% (67/68 animals positive, 95% CI = 89.83–99.80). On the other hand, only 51 of the 68 reactors were identified as positive by the SICCT test and hence, its relative sensitivity was 75.0% (95%CI = 63.1–84.1%). As a result, the relative sensitivity of the SICCT test was significantly (*p* < .001) lower than that of DSTc.

The diagnostic agreement of the DSTc and the SIT test as well as that of the DSTc and the SICCT test was evaluated on 107 cattle (68 TB reactor cows and 39 calves). Out of those cattle, 65 were DSTc-positive, while the remaining 42 were negative for the DSTc test (Table 1). The test agreement of the DSTc with the SIT and SICCT tests was 98.13% and 85.98%, respectively. Thus, a strong agreement (*k* = 0.96, *p* < .001) was recorded between the DSTc and SIT tests, while moderate agreement (*k* = 0.74, *p* < .001) was recorded between the DSTc and SICCT tests.

### 3.3 | Diagnostic performance of the DST fusion protein in reactor cattle

**FIGURE 2:**
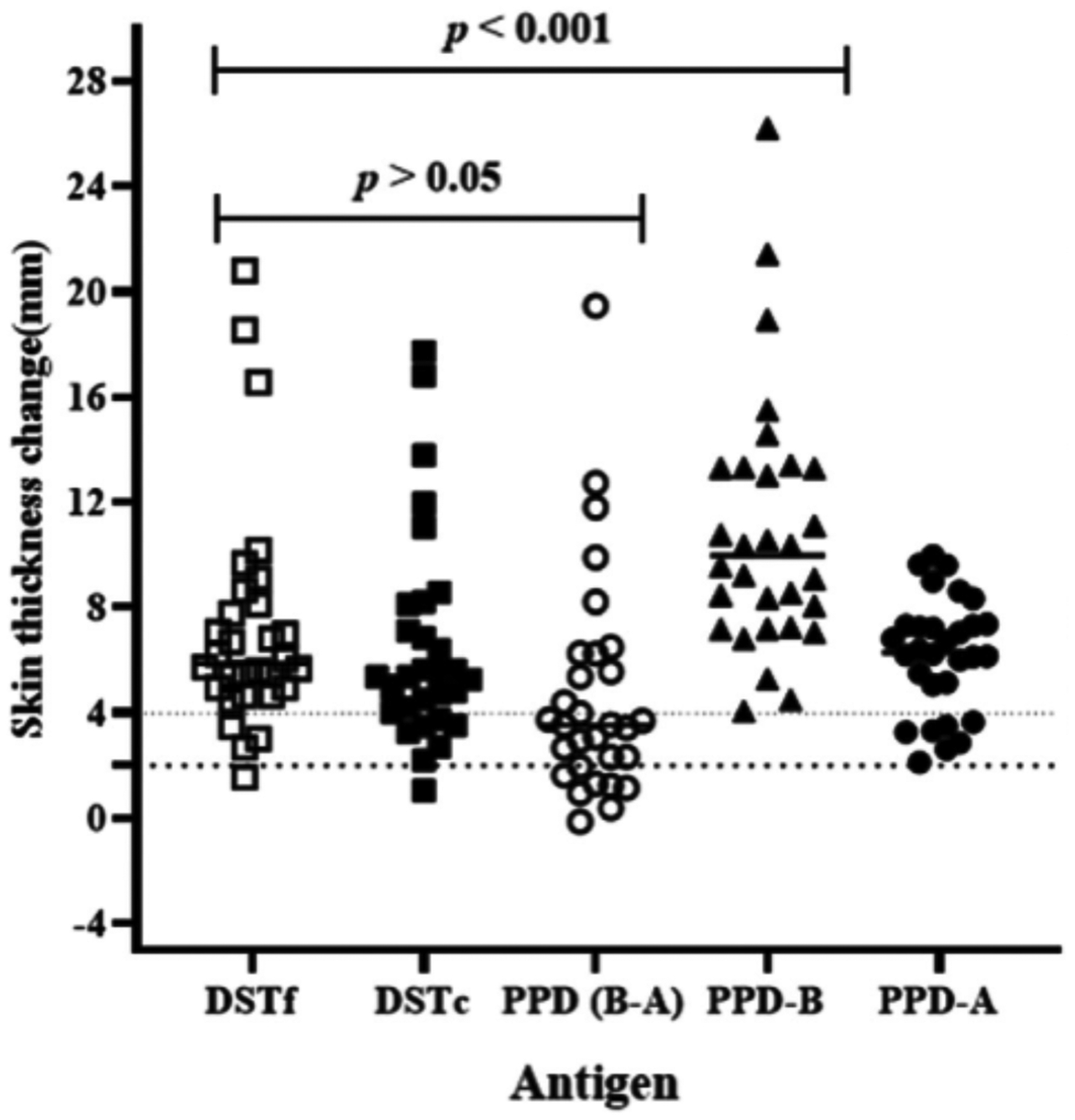
Comparison of skin reaction response of DSTf to the response of PPD-A PPD-B, PPD-(B-A) and that of DSTc in 30 naturally infected cows. The skin-fold thickness was measured before and after 72 h of injections. Results are shown as increases in skin thickness (mm), which are represented by open square, solid square, open circle, solid triangle and solid circle with horizontal lines providing the median skin thickness change of respective antigen. PPD-B induced significantly stronger skin reaction (*p* < .001) than that caused by DSTf using the Friedman test (repeated measures non-parametric analysis of variance) with Dunn’s multiple comparison tests. There was no significant difference (*p* > .05) between the responses induced by DSTf and DSTc in reactor cattle. The dashed horizontal lines at 2 and 4 mm are the cut-offs used for DSTf and DSTc, and PPD (B-A) and PPD-B, respectively

**FIGURE 3:**
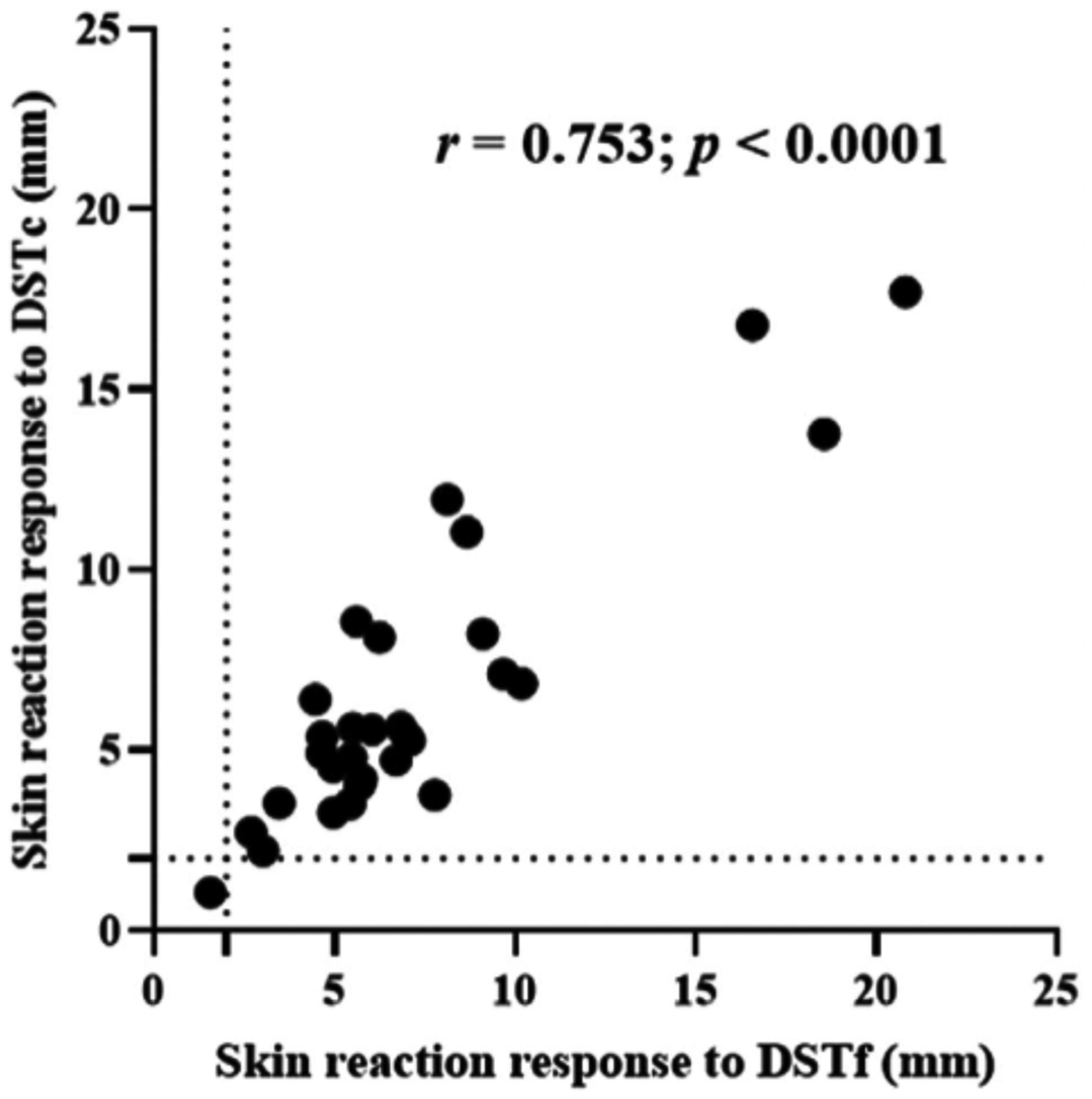
Correlation of skin reaction responses between the DSTf (dose 30 *μ*g) and the DSTc (dose 10 *μ*g per protein). Solid black circle represents an individual study animal. The dashed horizontal and vertical lines at 2 and 4 mm are the cut-offs used for DSTc and DSTf, respectively

The diagnostic performance of the DSTf was evaluated in 30 TB reactor cows. The reactivity of the skin to injection with DSTf was compared with the skin reactivity after injection with DSTc, PPD-B and PPD-A. The results of this experiment are presented in Figure 2. The median of skin thickness increases at the DSTf injection site was 5.89 mm (95% CI = 5.43–7.08) compared to 5.38 mm (IQR = 4.53–8.85) at the DSTc site. The medians of skin thicknesses were 6.32 mm (95% CI = 5.52–7.25) at the PPD-A site and 9.97 mm (IQR = 8.35–13.03) at the PPD-B site. Multiple comparison analysis revealed that the skin thickness caused by DSTf inoculation was statistically lower (*p* < .001) than that caused by PPD-B inoculation (Figure 2). On the other hand, the thickness caused by inoculation of DSTf was similar to the difference in skin thicknesses of the PPD-B site and PPD-A site (Figure 2, median difference between PPD-B and PPD-A readings was 3.55 mm, IQR = 3.34–5.39). Likewise, there was no significant difference (*p* > .05) in skin thicknesses caused by inoculation of DSTf and DSTc (Figure 2). A statistically, a strong correlation (*r* = 0.753; 95%CI = 0.532–0.879; *p* < .001) was recorded between the DSTf and DSTc as demonstrated in Figure 3.

## 4 | DISCUSSION

The present study was conducted to evaluate the performance of a DIVA skin test (DST), based on the *M. tuberculosis* complex proteins ESAT-6, CFP-10 and Rv3615c using either a cocktail (DSTc) or a fusion protein (DSTf), in differentiating *M. bovis*-infected and BCG-vaccinated cattle. The study was conducted on 74 non-infected calves, and 68 TB reactor cows from herd with confirmed bTB prevalence, and all cattle were Zebu–Holstein–Friesian crossbreed. The calves consisted of two groups, that is 35 BCG-vaccinates and 39 non-vaccinates. The results of the DSTc were analysed and evaluated for its performance as a DIVA skin test. Furthermore, the performance of the DSTc was compared with that of the SIT and SICCT tests in 68 TB reactor cows. Such evaluation of DIVA reagents has repeatedly been performed in taurine breed cattle in developed countries settings (Casal et al., 2012; Jones et al., 2012; Vordermeier et al., 2016a; Whelan et al., 2010, b). However, this is the first study to evaluate the DSTc and DSTf proteins in zebu-taurine crossbred cattle in the context of developing countries; hitherto, only a peptide cocktail of these antigens has been tested in TB reactor cattle in Ethiopia (Srinivasan et al., 2019).

The relative specificity of the DSTc was evaluated in 35 non-infected and BCG-vaccinated calves, which were recruited from known bTB free dairy herds and also re-affirmed to be free of bTB by IGRA. Six weeks after subcutaneous inoculation with 1×10^6^ CFU dose of BCG, they did not react to intradermal injection of the DSTc, giving a relative specificity of 100% (95% CI = 90.0–100). Similar to the present result, previous studies reported that antigenic protein- or peptide-based intradermal DSTc did not induce detectable skin reactions in BCG-vaccinated taurine cattle (Jones et al., 2012; Vordermeier et al., 2016b; Whelan et al., 2010). Similarly, all the 39 non-vaccinated calves did not react to all the three (DSTc, SIT and SICCT) tests and the specificity of DSTc in non-vaccinated calves was 100%. Furthermore, DSTc detected 65 of the 68 TB reactor cows, while the SICCT test detected 51 of them. Thus, the sensitivity of the DSTc was 95.6%, while the sensitivity of the SICCT test was 75%, suggesting the DSTc has higher sensitivity than the SICCT test. Recently, Srinivasan et al. (2019) recorded similar level of sensitivity of peptide cocktail-based DIVA skin test in bTB-positive cattle in Ethiopia, although our sample size was substantially larger. All recruited study animals were positive by the SIT as well as by the SICCT test during the evaluation trials. The sensitivity of the DSTc in the present study was higher than the sensitivity of the DSTc reported by other studies (Casal et al., 2012; Jones et al., 2012; Vordermeier et al., 2016a), here in crossbreed cattle, which encourages the future application of DSTc in conjunction with BCG usage.

In addition to estimation of the sensitivity and specificity of the DSTc, Kappa statistics was used to evaluate its agreement with the SIT and SICCT tests. The agreement between the DSTc and SIT tests was strong, while on the other hand, a moderate agreement was recorded between the DSTc and SICCT tests. In another field evaluation, a moderate agreement was recorded between the DSTc and SIT tests in 23 reactors (Casal et al., 2012). However, since the true disease status of the test animals was not known, it is difficult to make a conclusive remark on the sensitivities and specificities of DSTc unless a gold standard test (TB lesion and or *M. bovis* isolation) is used. Therefore, there is a need for further evaluation of the sensitivities and specificities of DSTc on a large number of cattle using the appropriate gold standard test.

With regard to the intensity of the skin thickness induced by the injection of the DSTc, the magnitude of skin thickness induced by DSTc and PPD-B was compared in TB reactor cows and it was observed that the median of skin thickness induced by DSTc injection (6.3 mm) was lower than those induced by PPD-B (10.8 mm). This observation agreed with the observations made earlier by other studies elsewhere in taurine cattle (Jones et al., 2012; Srinivasan et al., 2019; Whelan et al., 2010). The stronger skin reaction to PPD-B could be because it consists of a more diverse range of immunogenic proteins (Borsuk et al., 2009), whereas the DSTc contains only the three mycobacterial proteins ESAT-6, CFP-10 and Rv3615c. Moreover, a dose of tuberculin solution contains greater protein content than a dose of the DSTc (Yang et al., 2013). The strong skin reaction following PPD-B could also be due to less purification compared to the highly purified DSTc. Except Casal et al. (2012), who recorded comparable medians skin thicknesses by injection of DSTc and PPD-B, other researchers recommended the possibility of strengthening the skin reaction to DSTc by adding Rv3020 (Jones et al., 2012; Vordermeier et al., 2016b).

In most of the earlier studies, the experiments evaluating DIVA tests were conducted on whole blood-based IFN-*γ* assays for easiness of the protocol to accommodate modifications (Vordermeier et al., 2016b). However, the use of these DIVA reagents in the IFN-*γ* assay will be difficult to implement in areas with economic and technical constraints (Ameni et al., 2000). In contrast, the DIVA skin testing is a simpler technique and can easily be applied in the field in the same way as the tuberculin skin test. Thus, a recombinant fusion protein containing ESAT-6, CFP-10 and Rv3615-c (DSTf) was produced in a similar presentation as PPD-B, containing 1.2 ml (12 doses) per vial. In the present field trial in reactor cattle, the DSTc and DSTf demonstrated comparable medians of skin thickness in 30 TB reactor cows. Like DSTc, the DSTf induced skin reaction equivalent to the final skin thickness induced by the SICCT test. Therefore, these observations encourage the use of DSTc or DSTf for the diagnosis of bTB in cattle.

## 5 | CONCLUSION

This is the first study to investigate the performance of the DIVA skin test based on a cocktail/fusion protein of three mycobacterial antigens (ESAT-6, CFP-10 and Rv3615c) in zebu-taurine crossbred cattle. The data showed high sensitivity of the DSTc in TB reactor cows and its high specificity in BCG-vaccinated bTB free calves after 6 weeks of BCG vaccination. The data generated by the two DST preparations (cocktail and fusion) were comparable. Thus, the findings of this study demonstrated the potential utility of DSTc or DSTf to support BCG vaccine-based bTB control policies, although additional extended field evaluation of these tests is important for re-affirmation of the observations of this study.

## Supplementary Material

Supporting Information

## Figures and Tables

**TABLE 1:** Comparison of the diagnostic performance of the cocktail protein-based DIVA skin test (DSTc) with the performances of the single intradermal tuberculin (SIT) test and the single comparative cervical tuberculin (SICCT) test in detecting bTB infection

Skin test type	Evaluation of sensitivity in 68 TB reactor cows		
	No. of positive	No. of negative	% Sensitivity
DSTc	65	3	95.6 (87.6–99.1%)
SIT test	67	1	98.5 (92.1–100%)
SICCT test	51	17	75.0 (63.0–84.7%)

	Evaluation of test agreements in 107 cattle with different status of bTB					
		SIT test		SICCT test		
		Positive	Negative	Positive	Doubtful	Negative
DSTc	Positive	65	0	51	10	4
	Negative	2	40	0	1	41
	Total	**67**	**40**	**51**	**11**	**45**
Fischer’s exact *p* value		**.000**			**.000**	
Tests agreement (*k*)		**0.96**			**0.74**	

Bold values indicate *p* < 0.001.

## Data Availability

The raw data used for this study have been submitted with the manuscripts for availability for the researchers on the basis of request and acknowledgements.

## Abbreviations:

BCG: Bacillus Calmette–Guerin
BTB: bovine tuberculosis
CFP-10: culture filtrate protein 10
DIVA: differentiate infected from vaccinated
DST: DIVA skin test
DSTc: cocktail protein-based DST
DSTf: fusion protein-based DST
ESAT-6: early secretory antigen target 6 kDa
LMICs: low- and middle-income countries
PPD-A: avian purified protein derivative
PPD-B: bovine purified protein derivative
RD1: region of difference 1
SICCT: single intradermal cervical comparative tuberculin
SIT: single intradermal tuberculin

## References

[R1] Ameni G, Aseffa A, Engers H, Young D, Gordon S, Hewinson RG, & Vordermeier HM (2007). High prevalence and increased severity of pathology of bovine tuberculosis in Holsteins compared to zebu breeds under field cattle husbandry in central Ethiopia. Clinical and Vaccine Immunology, 14(10), 1356–1361. 10.1128/CVI.00205-07. 17761523 PMC2168113

[R2] Ameni G, Desta F, & Firdessa R (2010). Molecular typing of Mycobacterium bovis isolated from cattle slaughtered in northeastern Ethiopia. The Veterinary Record, 167, 138–141. 20656993 10.1136/vr.b4881

[R3] Ameni G, Miormer H, Roger F, & Tibbo M (2000). Comparison between comparative tuberculin and gamma-interferon test for the diagnosis of bovine tuberculosis in Ethiopia. Tropical Animal Health and Production, 32(5), 267–276. 10.1023/a:1005271421976. 11059035

[R4] Ameni G, Tafess K, Zewde A, Eguale T, Tilahun M, Hailu T, Sirak A, Salguero FJ, Berg S, Aseffa A, Hewinson RG, & Vordermeier HM (2018). Vaccination of calves with *Mycobacterium bovis* Bacillus Calmette–Guerin reduces the frequency and severity of lesions of bovine tuberculosis under a natural transmission setting in Ethiopia. Transboundary and Emerging Diseases, 65(1), 96–104. 10.1111/tbed14252.12618. 28168855 PMC5811905

[R5] Ashford DA, Whitney E, Raghunathan P, & Cosivi O (2001). Epidemiology of selected mycobacteria that infect humans and other animals. Scientific and Technical Review, 20(1), 325–337. 10.20506/rst.20.1.1266. 11288519

[R6] Bezos J, Casal C, Romero B, Schroeder B, Hardegger R, Raeber AJ, López L, Rueda P, & Domínguez L (2014). Current ante-mortem techniques for diagnosis of bovine tuberculosis. Research in Veterinary Science, 97, S44–S52. 10.1016/j.rvsc2014.04.002. 24768355

[R7] Borsuk S, Newcombe J, Mendum TA, Dellagostin OA, & McFadden J (2009). Identification of proteins from tuberculin purified protein derivative (PPD) by LC-MS/MS. Tuberculosis, 89(6), 423–430. 10.1016/j.tube.2009.07.003. 19683472

[R8] Buddle BM, Livingstone PG, & de Lisle GW, (2009). Advances in antemortem diagnosis of tuberculosis in cattle. New Zealand Veterinary Journal, 57(4), 173–180. 10.1080/00480169.2009.36899. 19649010

[R9] Buddle BM, Palane NA, Wedlock DN, & Heiser A (2013). Overview of vaccination trails for control of tuberculosis in cattle, wildlife and humans. Transboundary and Emerging Diseases, 60(2), 136–146. 10.1111/tbed14252.12092. 24171859

[R10] Buddle BM, Vordermeier MH, Chambers MA, & de Klerk-Lorist LM (2018). Efficacy and safety of BCG vaccine for control of tuberculosis in domestic livestock and wildlife. Frontier in Veterinary Sciences, 5, 1–17, www.frontiersin.org 10.3389/fvets.2018.00259 PMC6214331 30417002

[R11] Casal C, Bezos J, Diez-Guerrier A, Alvarez J, Romero B, de Juan L, Rodriguez-Campos S, Vordermeier M, Whelan A, Hewinson RG, Mateos A, Dominguez L, & Aranaz A (2012). Evaluation of two cocktail containing ESAT-6, CFP-10 and Rv-3615c in the interdermal test and the interferon-Y assay for diagnosis of bovine tuberculosis. Preventive Veterinary Medicine, 105, 149–154. 10.1016/j.prevetmed.2012.02.007. 22391021

[R12] de la Rua-Domenech R, Goodchild AT, Vordermeier HM, Hewinson RG, Christiansen KH, & Clifton-Hadley RS (2006). Ante mortem diagnosis of tuberculosis in cattle: a review of the tuberculin tests, gamma-interferon assay and other ancillary diagnostic techniques. Research in Veterinary Science, 81(2), 190–210. doi: 10.1016/j.rvsc.2005.11.005. 16513150

[R13] Firdessa R, Tschopp R, Wubete A, Sombo M, Hailu E, Erenso G, Kiros T, Yamuah L, Vordermeier HM, Hewinson RG, Young D, Gordon SV, Sahle M, Aseffa A, & Berg S (2012). High prevalence of bovine tuberculosis in dairy cattle in Central Ethiopia: Implications for the dairy industry and public health. PLoS One, 7(12), e52851. 10.1371/journal.pone.0052851. 23285202 PMC3532161

[R14] Garnier T, Eiglmeier K, Camus JC, Medina N, Mansoon H, Pryor M, Duthoy S, Grondin S, Lacroix C, Monsempe C, Simon S, Harris B, Atikin R, Doggett J, Mayes R, Keating L, Wheeler PR, Parkhill J, Barrell BG, … Hewinson RG (2003). The complete genome sequence of *Mycobacterium bovis*. Proceedings of the National Academy of Sciences of the United States of America, 100(13), 7877–7882. 10.1073/pnas.1130426100. 12788972 PMC164681

[R15] Gordon SV, Brosch R, Billault A, Garnier T, Eiglmeier K, & Cole ST (1999). Identification of variable regions in the genomes of tubercle bacilli using bacterial artificial chromosome arrays. Molecular Microbiology, 32(3), 643–655. 10.1046/j.1365-2958.1999.01383.x. 10320585

[R16] Jones GJ, Whelan A, Clifford D, Coad M, & Vordermeier HM (2012). Improved skin test for differential diagnosis of bovine tuberculosis by the addition of Rv3020c-derived peptide. Clinical and Vaccine Immunology, 19(4), 620–622. 10.1128/CVI.00024-12. 22301696 PMC3318272

[R17] Mekonnen GA, Conlan AJK, Berg S, Ayele BT, Alemu A, Guta S, Lakew M, Tadesse B, Gebre S, Wood JLN, & Ameni G, & The ETHICOBOTS Consortium. (2019). Prevalence of bovine tuberculosis and its associated risk factors in the emerging dairy belts of regional cities in Ethiopia. Preventive Veterinary Medicine, 168, 81–89. 10.1016/j.prevetmed.2019.04.010. 31097127 PMC10364076

[R18] Pollock JM, & Anderson P (1997). The potential of the ESAT-6 antigen secreted by virulent mycobacteria for specific diagnosis of tuberculosis. Journal of Infectious Diseases, 175(5), 1251–1254. 10.1086/593686. 9129098

[R19] OIE. (2018). Terrestrial manual: Bovine tuberculosis. Paris: World Organization for Animal Health Press.

[R20] Schiller I, Oesch B, Vordermeier HM, Palmer MV, Harris BN, Orloski KA, Buddle BM, Thacker TC, Lyashchenko KP, & Waters WR (2010). Bovine tuberculosis: A review of current and emerging diagnostic techniques in view of their relevance for disease control and eradication. Transboundary and Emerging Diseases, 57(4), 205–220. 10.1111/j.1865-1682.2010.01148x. 20561288

[R21] Sibhat B, Asmare K, Demissie K, Ayelet G, Mamo G, & Ameni G (2017). Bovine tuberculosis in Ethiopia: A systematic review and meta-analysis. Preventive Veterinary Medicine, 147, 149–157. 10.1016/j.prevetmed.2017.09.006. 29254713 PMC5739073

[R22] Sidders B, Pirson C, Hogarth PJ, Hewinson RG, Stoker NG, Vordermeier HM, & Ewer K (2008). Screening of highly expressed mycobacterial genes identifies Rv3615c as a useful differential diagnostic antigen for the *Mycobacterium tuberculosis* complex. Infection and Immunity, 76(9), 3932–3939. 10.1128/IAI.00150-08. 18519559 PMC2519431

[R23] Srinivasan S, Jones G, Veerasami M, Steinbach S, Holder T, Zewude A, Fromsa A, Ameni G, Easterling L, Bakker D, Juleff N, Gifford G, Hewinson RG, Vordermeier HM, & Kapur V (2019). A defined antigen skin test for the diagnosis of bovine tuberculosis. Science Advances, 5, aax4899. 10.1126/sciadv.aax4899. PMC6636981 31328169

[R24] Tschopp R, Hattendorf J, Roth F, Choudhoury A, Shaw A, Aseffa A, & Zinsstag J (2012). Cost estimation of bovine tuberculosis to Ethiopia. Current Topics in Microbiology and Immunology, 365, 249–268. 10.1007/82_2012_245. 22806204

[R25] Vordermeier HM, Gordon SV, & Hewinson RG (2011). *Mycobacterium bovis* antigens for the differential diagnosis of vaccinated and infected cattle. Veterinary Microbiology, 151, 8–13. 10.1016/j.vetmic.2011.02.020. 21411245

[R26] Vordermeier HM, Jones GJ, Buddle BM, & Hewinson RG (2016a). Development of immune-diagnostic reagents to diagnose bovine tuberculosis in cattle. Veterinary Immunology and Immunopathology, 181, 10–14. 10.1016/j.vetimm.2016.02.003. 26906942

[R27] Vordermeier HM, Jones GJ, Buddle BM, Hewinson RG, & Villarreal-Ramos B (2016b). Bovine tuberculosis in cattle: Vaccine, DIVA test, and host biomarker discovery. Annual Review of Animal Biosciences, 4, 87–109. 10.1146/annurev-animal-021815-111311. 26884103

[R28] Vordermeier HM, Chambers MA, Cockle PJ, Whelan AO, Simmons J, & Hewinson RG (2002). Correlation of ESAT-6-specific gamma interferon production with pathology in cattle following *Mycobacterium bovis* BCG vaccination against experimental bovine tuberculosis. Infection and Immunity, 70(6), 3026–3032. 10.1128/iai.70.6.3026-3032.2002. 12010994 PMC128013

[R29] Vordermeier HM, Cockle PC Whelan AO, Rhodes S, Palmer N, Bakker D, & Hewinson RG (1999). Development of diagnostic reagents to differentiate between *Mycobacterium bovis* BCG vaccination and *M. bovis* infection in cattle. Clinical and Diagnostic Laboratory Immunology, 6(5), 675–682. https://doi.org/1071-412X/99/＄04.0010 10473516 10.1128/cdli.6.5.675-682.1999 PMC95753

[R30] Vordermeier HM, Villarreal-Ramos B, Cockle PJ, McAulay M, Rhodes SG, Thacker T, Gilbert SC, McShane H, Hill AV, Xing Z, & Hewinson RG (2009). Viral booster vaccines improve *Mycobacterium bovis* BCG-induced protection against bovine tuberculosis. Infection and Immunity, 77(8), 3364–3373. 10.1128/IAI.00287-09. 19487476 PMC2715681

[R31] Vordermeier HM, Whelan A, Cockle PJ, Farrant L, Palmer N, & Hewinson RG (2001). Use of synthetic peptides derived from the antigens ESAT-6 and CFP-10 for differential diagnosis of bovine tuberculosis in cattle. Clinical and Diagnostic Laboratory Immunology, 8(3), 571–578. 10.1128/CDLI.8.3.571-578.200. 11329460 PMC96103

[R32] Whelan OA, Clifford D, Upadhyay B, Breadon LE, McNair J, Hewinson RG, & Vordermeier HM (2010). Development of a skin test for bovine tuberculosis for differentiating infected from vaccinated animals. Journal of Clinical Microbiology, 48(9), 3176–3181. 10.1128/JCM.00420-10. 20592155 PMC2937719

[R33] Yang H, Kruh-Garcia NA, & Dobos KM (2013). Purified protein derivatives of tuberculin—Past, present, and future. FEMS Immunology and Medical Microbiology, 66(3), 273–280. 10.1111/j.1574-695X.2012.01002.x. PMC3491170 22762692

